# *Inula japonica* Thunb. Flower Ethanol Extract Improves Obesity and Exercise Endurance in Mice Fed a High-Fat Diet

**DOI:** 10.3390/nu11010017

**Published:** 2018-12-20

**Authors:** So-Hyun Park, Da-Hye Lee, Min Jung Kim, Jiyun Ahn, Young-Jin Jang, Tae-Youl Ha, Chang Hwa Jung

**Affiliations:** 1 Department of Food Biotechnology, Korea University of Science and Technology, Wanju-gun, Jeollabuk-do 55365, Korea; Park.So-hyun@kfri.re.kr (S.-H.P.); Lee.Da-hye@kfri.re.kr (D.-H.L.); jyan@kfri.re.kr (J.A.); tyhap@kfri.re.kr (T.-Y.H.); 2 Research Division of Food Functionality, Korea Food Research Institute, Wanju-gun, Jeollabuk-do 55365, Korea; kmj@kfri.re.kr (M.J.K.); jyj616@kfri.re.kr (Y.-J.J.)

**Keywords:** *Inula japonica*, obesity, exercise endurance, adipogenesis, lipogenesis, myogenesis

## Abstract

*Inula japonica* Thunb. (Asteraceae) is a flowering plant that grows mainly in Korea, Japan, and China and its flower extract has diverse biological effects such as anti-inflammatory and antioxidative activities. However, the effects on obesity and enhancement of endurance capacity have not been explored yet. This study aims to reveal the effects of *I. japonica* flower ethanol extract (IJE) on obesity and endurance capacity in high-fat diet (HFD) fed C57BL/6J mice and the mechanism. IJE inhibited lipid accumulation in 3T3-L1 adipocytes in vitro. Also, IJE-fed mice showed reduced body weight gain, hepatic lipid, and body fat mass, and increased muscle weight. IJE reduced lipid accumulation in the liver and adipose tissue by decreasing lipogenic and adipogenic gene expression. Additionally, consumption of low-dose IJE significantly enhanced endurance capacity via increasing AMP-activated protein kinase activity and mRNA levels of *Myh7* and *Myh2*. Luteolin and 1β-hydroxyalantolactone (1β-HA), compounds of IJE, are involved in anti-adipogenesis in the 3T3-L cells and only luteolin increased the protein levels of MHC during C2C12 myoblast differentiation. Collectively, our results suggest that consumption of IJE not only helps to prevent obesity but also enhances endurance capacity reduced by HFD.

## 1. Introduction

*Inula* (Asteraceae) is a large genus containing about 100 species of flowering plants that grow in Asia, Africa, Europe, and Mediterranean countries. Among them, 20 species are distributed in Northern Asia [1]. Inula species are a rich source of sesquiterpenoids, which are reported to exhibit biological activities such as anti-inflammatory, anti-tumor, and anti-angiogenic activities [2,3,4].

*Inula japonica* Thunb. (Asteraceae) is mainly distributed in Korea, Japan, and northern China. It is called geumbulcho in Korea and used as a folk medicine to control asthma, coughs, and phlegm. Previous studies have shown that *I. japonica* extract attenuates mast cell-mediated allergic reaction in mice sensitized to immunoglobulin E [5]. Polysaccharides extracted from *I. japonica* exhibit anti-diabetic activity in mice with alloxan or streptozotocin-induced diabetes by protecting β-cells and decreasing blood glucose level and oxidative stress [6,7,8]. Additionally, essential oils extracted from *I. japonica* increase the sensitivity of MCF-7/ADR cells to doxorubicin by downregulating the expression of ATP-binding cassette sub-family B member 1. They have also been suggested to be effective multidrug resistance reversal agents [9]. *I. japonica* contains various dimeric sesquiterpene lactones, which include japonicones and neojaponicones [10,11,12]. Several sesquiterpenes significantly inhibit lipopolysaccharide-induced nitric oxide production in RAW 264.7 macrophages [13,14]. Furthermore, other compounds isolated from *I. japonica*, such as luteolin, quercetagetin, 3,4-dimethyl ether, britanin, and tomentosin, have been shown to potently inhibit topoisomerase activity and cytotoxicity in A549 and HT-29 cells [15].

Although *I. japonica* has various biological activities, its effects on obesity and exercise capacity have not been evaluated yet. In this study, we investigated the effects of the *I. japonica* flower ethanol extract (IJE) on adipocyte differentiation in 3T3-L1 cells. Additionally, the anti-obesity and endurance capacity enhancement effects of the extract in mice with high-fat diet (HFD)-induced obesity were investigated.

## 2. Materials and Methods

### 2.1. Reagent

Dulbecco’s Modified Eagle’s Medium (DMEM), fetal bovine serum (FBS), and calf serum (CS) were purchased from HyClone (Logan, UT, USA). IBMX (I7018), dexamethasone (D4902), insulin (I0908) and Oil Red O powder (O0625) were purchased from Sigma-Aldrich (Saint Louis, MO, USA). Horse serum (HS), penicillin-streptomycin (PS) 100× solution, penicillin/streptomycin/glutamine (PSG) 100× solution, protease and phosphatase inhibitor cocktails, radioimmunoprecipitation assay buffer (RIPA) buffer, and enhanced chemiluminescence (ECL) western substrate were purchased from Thermo Fisher Scientific (Waltham, MA, USA).

### 2.2. Preparation of I. japonica Flower Ethanol Extract (IJE)

Dried *I. japonica* flowers from China were purchased from Mi-Ryong herbal medicine co. (No. SBH 141111-01; Seoul, Korea) in March 2017. A voucher specimen (JCH No.21) was deposited at the Korea Food Research Institute (Wanju-gun, Jeollabuk-do, Korea). The flower was identified by Professor Seong-Gyu Ko (Department of Preventive Medicine, Kyung Hee University, Seoul, Korea). The plant was pulverized using a mill, after which the powder was subjected to extraction twice at 80 °C for 2 h with 10 times the volume of 70% ethanol. The extract was filtered and concentrated using an evaporator. Finally, the extract was freeze-dried and stored at −20 °C until use.

### 2.3. Differentiation and Oil Red O Staining in 3T3-L1 Cells

3T3-L1 cells were purchased from American Type Culture Collection (Manassas, VA, USA). The cells were cultured in high-glucose DMEM supplemented with 10% CS and PSG, and seeded into a 6-well plate (4 × 10^5^ cells/well). After 2 days of seeding the cells, the culture medium was changed to differentiation medium (DMEM containing 10% FBS, 0.5 mM IBMX, 1 µM dexamethasone, and 1 μg/mL insulin). After 30 min, the cells were treated with IJE and incubated for 2 days. The medium was then replaced with DMEM containing 10% FBS and 1 μg/mL insulin and incubated for 2 days. Finally, the medium was replaced with DMEM containing 10% FBS until differentiation was terminated. Oil Red O staining of differentiated 3T3-L1 cells was performed as preciously described method [16] and all 3T3-L1 cell experiment was performed in triplicate experiments.

### 2.4. Cell Viability

The cells were seeded and incubated in 96-well plates (1 × 10^4^ cells/well). Next day, the cells were treated with 0–200 μg/mL of IJE. After 24 h, 20 μL of 5 mg/mL MTT (Sigma, Saint Louis, MO, USA) in phosphate-buffered saline (PBS) was added to each well, followed by incubation of the plates for 2 h. Thereafter, all media were removed, and dimethyl sulfoxide was added to the cells. Absorbance was measured at 540 nm using a microplate reader (Infinite M200; Tecan US, Inc., Morrisville, NC, USA).

### 2.5. Quantitative Reverse Transcription Polymerase Chain Reaction (PCR)

RNA was extracted using RNeasy Mini Kit and RNeasy Fibrous Tissue Mini Kit (Qiagen, Inc., Valencia, CA, USA). All cDNA was synthesized using ReverTra Ace^®^ quantitative reverse transcription polymerase chain reaction (qPCR RT) kit (Toyobo Co., Ltd., Osaka, Japan) according to the manufacturer’s instructions. After cDNA synthesis, quantitative PCR was performed using SYBR Green real-time PCR Master Mix (Toyobo Co., Ltd.) and StepOnePlus Real-Time PCR system (Applied Biosystems; Thermo Fisher Scientific, Inc., Foster City, CA, USA). Relative RNA levels were calculated after normalization of values to those of *Actb*(*β-actin*) or *Rn18s* (muscle RNA only) mRNA. The primer sequences are shown in Table 1.

### 2.6. Immunoblotting

Cells and tissues were harvested using RIPA buffer supplemented with protease and phosphatase inhibitor cocktails. The supernatants were harvested after centrifuging at 16,000 × *g* for 10 min at 4 °C. Proteins were separated by sodium dodecyl sulfate-polyacrylamide gel electrophoresis and transferred onto membranes. The membranes were blocked with 5% skimmed milk for 1 h and incubated overnight at 4 °C with the primary antibodies; Anti-peroxisome proliferator-activated receptor (PPAR)-γ (sc-7196, 1:1000) anti-CCAAT-enhancer-binding protein α (C/EBPα) (sc-7962, 1:1000), anti-β-actin (sc-47778, 1:1000), anti-glyceraldehyde 3-phosphate dehydrogenase (GAPDH) (sc-25778, 1:1000) (Santa Cruz, CA, USA), anti-adipocyte protein 2 (2120S, 1:1000), anti-fatty acid synthase (FAS) (3180, 1:1000), anti-phosphorylated-ADP-activated kinase α (AMPK α) (2535, 1:1000), anti-AMPKα antibodies (2793, 1:1000) (Cell signaling technology, Danvers, MA, USA) and total myosin heavy chain (MHC, MF-20, 0.5 μg/mL) (Developmental Studies Hybridoma Bank, Iowa City, IA, USA). The next day, the membranes incubated with secondary antibodies (NOVUS, CO, USA) dissolved in 5% skimmed milk for 1 h at room temperature. Protein signal was visualized using G:BOX Chemi XX6 (Syngene Ltd., Frederick, MD, USA) with ECL western substrate. Band density was determined using Image J software (National Institutes of Health, Bethesda, MD, USA).

### 2.7. Animal Care and Diet Composition

Male C57BL/6J mice (4-week-old) were purchased from Japan SLC, Inc. (Hamamatsu, Japan) for the study. The mice were housed in cages (*n* = 2 per cage) under a 12/12 h light/dark cycle at 22 ± 2 °C. After a week of adaptation, the animals were divided into four groups (*n* = 6 per group): Normal diet (N), HFD, HFD+IJE 0.25% (HFD+IL), and HFD+IJE 0.5% (HFD+IH). The diet compositions are shown in Table 2. Body weight was measured weekly, whereas food intake was measured every 2 days for 9 weeks. All in vivo studies were conducted in accordance with institutional and national guidelines. The protocol for the study was approved by the Korea Food Research Institute Animal Care and Use Committee (KFRI-M-17007 ER160900-01).

### 2.8. Body Composition Analysis by Dual-Energy X-ray Absorptiometry (DEXA)

Whole body image, fat mass, and lean body mass were obtained by performing dual-energy X-ray absorptiometry (DEXA) with InAlyzer (Medikors Co., Seongnam, Korea). The mice were anesthetized, placed on the scanner bed, and then scanned according to the instructions for operating the InAlyzer system.

### 2.9. Triglyceride (TG) and Total Cholesterol (TC) Levels and Staining with Hematoxylin and Eosin (H&E)

Serum triglyceride (TG) and total cholesterol (TC) levels were measured using slide kits (TG; 1650, TC; 1450) and Fuji dry-chem 3500s (Fuji film Co., Tokyo, Japan). Hepatic TG (20186), and TC (20081) levels were measured using the commercial kits (Shinyang Chemical Co., Busan, Korea) after extracting samples by the method described by Folch [17]. Liver and epididymal fat pad tissues were fixed in 4% formaldehyde, embedded in paraffin, cut into 5-μm sections, and stained with H&E. The samples were then observed under a microscope (IX71, Olympus Co., Tokyo, Japan). Adipose tissue sizes were measured using i-Solutions DT software.

### 2.10. Measurement of Exercise Capacity

The treadmill test was conducted for 3 days. On the first and second days, all mice were adapted to the treadmill running machine through training for 20 min at a 10° incline (day 1, 5 m/min for 10 min and 10 m/min for 10 min; day 2, 5 m/min for 5 min and 10 m/min for 15 min). On the final day, the mice ran at 10 m/s for the first 10 min, after which the speed was increased by 2 m/min every 2 min. The endpoint was set at when the mice did not move for 10 s. The results of the experiment were converted to distance. Grip strength was measured five times using a grip strength test machine (model GT3; Bioseb, FL, USA) according to the manufacturer’s instructions and the result was standardized by body weight. The average value was calculated, whereas the maximum and minimum values were not.

### 2.11. Liquid Chromatography–Mass Spectrometry (LC–MS/MS) Analysis

The analyses were performed using an Acquity ultra high-performance liquid chromatography (UPLC) system (Waters Co., Miliford, MA, USA) with Acquity UPLC BEH C18 column (2.1 mm × 100 mm, 1.7 µm). The mobile phase included 0.1% formic acid aqueous solution (Solvent A) and 0.1% formic acid in acetonitrile (Solvent B) and a gradient elution program was performed: 0–10 min, 99–70% solvent A; 10–11 min, 70–5% solvent A; 11–12 min, 5–99% solvent A; 12–13 min, 99% solvent A. The flow rate was set at 0.65 m/min and column temperature was kept at 40 °C. The auto-sampler was conditioned at 4 °C and the injection volume was 5 µL. Mass spectrometric analyses were operated using a Waters Xevo TQ triple-quadrupole mass spectrometer (Waters Co.) equipped with electrospray ionization (ESI) mode. The ESI source was operated switching between positive and negative ion mode with multiple reaction monitoring (MRM) mode. Data processing was performed using Progenesis QI software (Nonlinear Dynamics Ltd., Newcastle, UK) for chromatographic alignment, normalization, peak picking, and compound identification. The quantification was performed using negative mode of m/z 249.1→231.2 for 1β-hydroxyalantolactone (1β-HA) and positive mode of m/z 285.1→133.1 for luteolin. The detector was operated in cone voltage 30 V, a capillary voltage 3.0 kV. The source temperature was set at 150 °C, while the desolvation flow was set at 800 L/h, the desolvation gas temperature was set at 400 °C. Luteolin (L9283) and 1β-HA (CFN92600) used to analyze were purchased from Sigma-Aldrich and Chemfaces (Wuhan, China) respectively.

### 2.12. Immunofluorescence Study

The C2C12 cells were cultured in DMEM containing 10% FBS and PS, after which they were seeded into a 6-well plate (2 × 10^5^ cells/well) for differentiation. When confluency reached 90%, the medium was replaced every day with DMEM containing 2% horse serum (HS) for 3 days. Differentiated C2C12 cells were washed and fixed with 4% formaldehyde for 30 min. Next, the cells were permeabilized with 0.1% Triton X-100 in phosphate-buffered saline (PBS) for 10 min and blocked with 3% bovine serum albumin in 0.1% Triton X-100 in PBS for 1 h. The cells were then incubated overnight with total MHC antibody in blocking buffer at 4 °C and stained with Alexa Fluor 488-conjugated secondary antibody (Cell Signaling Technology) and 4′,6-diamidino-2-phenylindole. Images were captured using a fluorescent microscope (IX71, Olympus Co.). Nuclei within myotubes were counted at least 1000 nuclei per group.

### 2.13. Statistical Analysis

Differences among groups were evaluated using GraphPad Prism 7 software (GraphPad Software, Inc., San Diego, CA, USA). The statistical significant was determined one-way analysis of variance (one-way ANOVA) and Tukey’s multiple comparison test was used as a post hoc test (*p* < 0.05). Data are presented as mean ± standard deviation (SD) or mean ± standard error of the mean (SEM).

## 3. Results

### 3.1. IJE Inhibits Adipogenic Differentiation of 3T3-L1 Cells

**Figure 1:**
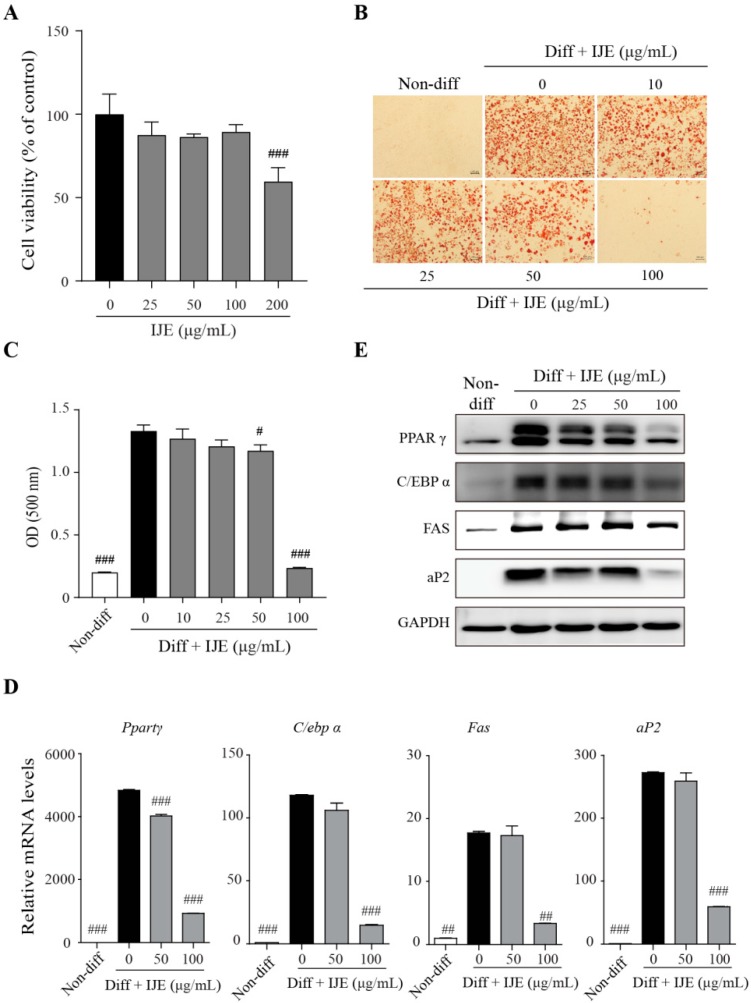
Effects of *I. japonica* flower ethanol extract (IJE) on adipogenic differentiation of 3T3-L1 cells. (**A**) The effects of IJE on cell viability were measured by the MTT assay. (**B**) Oil Red O staining of differentiated 3T3-L1 cells with or without IJE treatment was done (scale bar: 100 μm), after which absorbance was measured at 500 nm in triplicate. (**C**) Optical density (OD). (**D**) Relative mRNA and (**E**) protein levels of PPARγ, C/EBPα, FAS, and aP2. Data are presented mean ± standard deviation (SD) of triplicate experiments. #, ##, and ### indicate *p* < 0.05, 0.01, and 0.001, respectively, when compared to the group treated with differentiation (Diff) without IJE.

We examined the effects of IJE on lipid accumulation in 3T3-L1 cells. To investigate the cytotoxicity of IJE, 3T3-L1 cells were treated with different concentrations (0–200 μg/mL) of IJE for 24 h. The results showed that cell viability was not significantly affected at concentrations less than 100 μg/mL (Figure 1A). Differentiation of 3T3-L1 cells was investigated with 0–100 μg/mL IJE and the degree was measured by Oil Red O staining. The results showed that Oil Red O-stained areas were dose-dependently fewer at IJE concentrations of 50 and 100 μg/mL (Figure 1B,C). Furthermore, the mRNA and protein expression levels of PPARγ, aP2, FAS, and C/EBPα were reduced in 100 μg/mL IJE-treated adipocytes (Figure 1D,E), which indicate that IJE suppresses adipogenic differentiation of 3T3-L1 cells by inhibiting the mRNA and protein expression of the adipogenesis related genes.

### 3.2. IJE Prevents Increase in Body Weight

**Figure 2:**
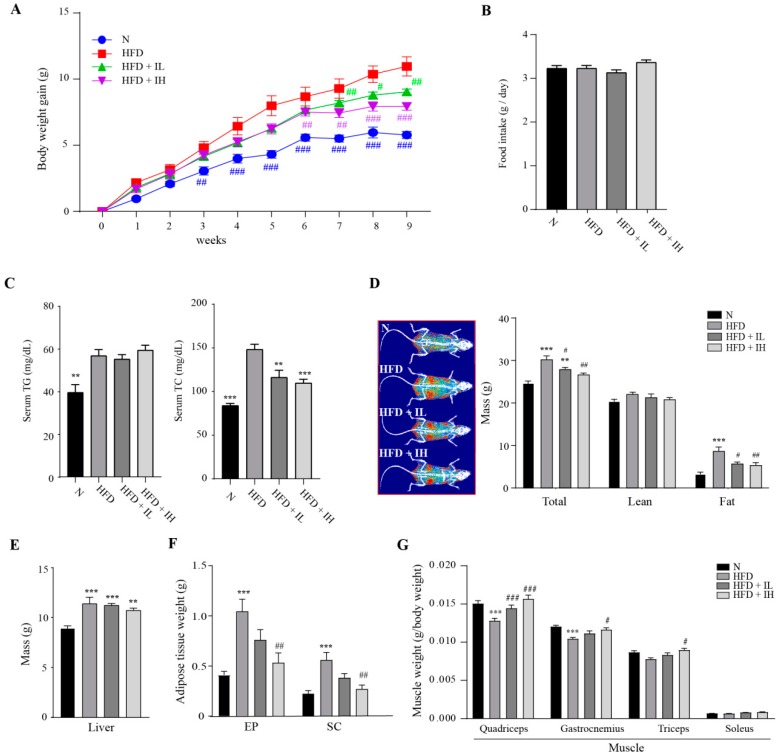
Effects of IJE on body weight and composition in high-fat diet (HFD)-induced obesity mice. (**A**) Body weight gain in mice fed different diets for 9 weeks (*n* = 6). (**B**) Mean daily food intake. (**C**) Serum TG and TC level. (**D**) Body composition image and bar chart showing total, lean, and fat body masses measured by dual-energy X-ray absorptiometry (DEXA) (*n* = 4). (**E**) Liver, (**F**) adipose tissue, and (**G**) muscle weights (g/body weight). Data are presented as mean ± standard error of the mean (SEM). ** indicates *p* < 0.01, whereas *** indicates *p* < 0.001 when compared to the N group. # indicates *p* < 0.05, ## indicates *p* < 0.01, and ### indicates *p* < 0.001 when compared to the HFD group. HFD, high-fat diet; HFD+IL, HFD+IJE 0.25% of diet; HFD+IH, HFD+IJE 0.5% of diet; N, normal diet.

The in vivo effects of IJE on fat accumulation and body composition were investigated for 9 weeks. The mice of experimental groups were fed two doses of IJE; low dose: 0.25% of diet; high dose: 0.5% of diet. The body weight gain was significantly lower in IJE-treated groups than it was in the HFD group without difference of food intake between the groups (Figure 2A,B). Also, IJE reduced serum TC level although the TG level was not different (Figure 2C). The body composition analysis by DEXA showed that fat body mass decreased in the IJE-treated groups (Figure 2D). Additionally, liver weight was slightly reduced (Figure 2E). Adipose tissue weight was more dramatically decreased in the IJE group than it was in the HFD group (Figure 2F). Furthermore, muscle weight (g/body weight) decreased by HFD was recovered by IJE treatment (Figure 2G). These results suggest that IJE reduces body fat mass but increases muscle weight.

### 3.3. IJE Improves Abnormal Lipid Accumulation in the Liver and Adipose Tissue

**Figure 3:**
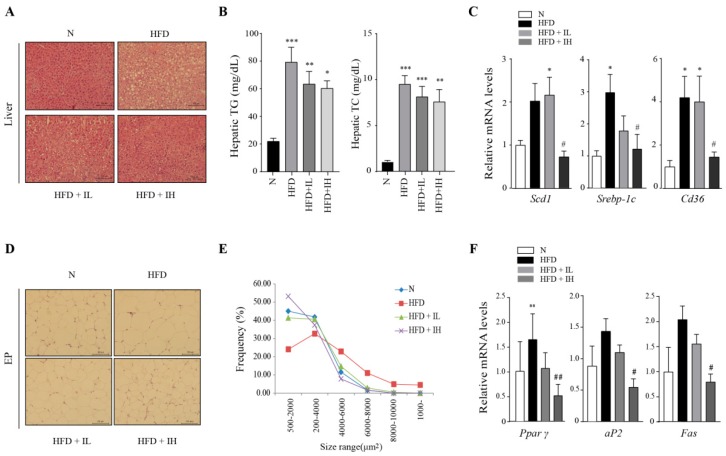
Effects of IJE on lipid accumulation in the liver and adipose tissue. (**A**) Images showing hematoxylin and eosin (H&E)-stained cross-sections of the liver (scale bar: 100 μm). (**B**) Hepatic triglyceride (TG) and total cholesterol (TC) levels. (**C**) Relative mRNA levels of *Scd1*, *Srebp-1c,* and *Cd36* in the liver. (**D**) Images of H&E-stained cross-sections of epididymal fat pad (scale bar: 100 μm). (**E**) Frequency of average cell size (*n* = 3) in epididymal fat pad. (**F**) Relative mRNA levels of *Pparγ*, *aP2*, and *Fas* in epididymal fat pad. Data are presented as mean ± SEM. *, **, and *** indicate *p* < 0.05, 0.01, and 0.001, respectively, when compared to the N group. # indicates *p* < 0.05, whereas ## indicates *p* < 0.01 when compared to the HFD group. HFD, high-fat diet; HFD+IL, HFD+IJE 0.25% of diet; HFD+IH, HFD+IJE 0.5% of diet; N, normal diet.

Lipid mainly accumulates in adipocytes, however, continuous consumption of HFD can lead to fat accumulation in the liver. Furthermore, it can lead to the development of non-alcoholic fatty liver disease [18]. Analysis of liver cross-sections by H&E staining revealed that fat accumulation occurred in the livers of the HFD mice; however, supplementation with IJE decreased the size and number of lipid droplets in the liver (Figure 3A). Likewise, hepatic TG and TC levels slightly decreased in the IJE-treated groups; however, they were not significantly different from their respective levels in the HFD group (Figure 3B). We also investigated the effects of IJE on the expression of lipogenic genes. The results showed that the mRNA levels of *stearoyl-CoA desaturase-1* (*Scd1*), *cluster of differentiation 36* (*Cd36*) and *sterol regulatory element-binding protein 1c* (*Srebp-1c*) were significantly reduced in the HFD+IH group (Figure 3C). Similarly, the sizes of adipose cells were decreased in the IJE-treated groups (Figure 3D,E). Furthermore, the expression levels of *Pparγ, aP2,* and *Fas* were reduced by IJE (Figure 3F). Taken together, the results indicate that IJE suppresses HFD-induced abnormal lipid accumulation in the liver and adipose tissue.

### 3.4. IJE Enhances Muscle Endurance Capacity

**Figure 4:**
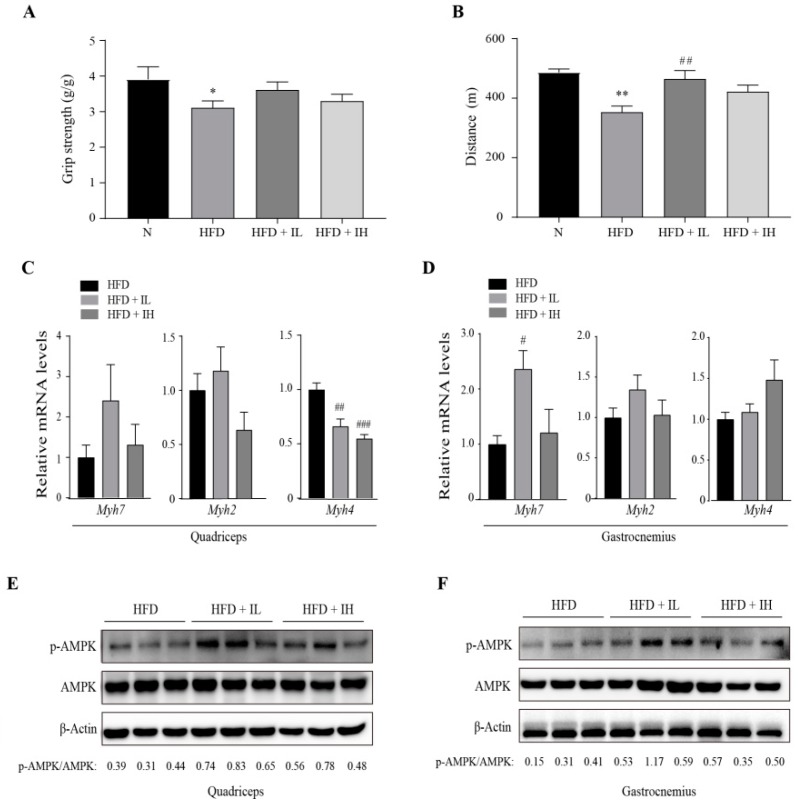
Effects of IJE on muscle exercise endurance capacity in HFD-induced obesity mice. Results of the (**A**) grip strength and (**B**) treadmill tests. (**C**,**D**) Relative mRNA expression levels of *Myh7*, *Myh2* and *Myh4* in the quadriceps and gastrocnemius muscles (*n* = 6). (**E**,**F**) Protein level of phosphorylated AMPK in the muscles. Data are presented as mean ± SEM. * and ** indicate *p* < 0.05 and 0.01, respectively, when compared to the N group. # indicates *p* < 0.05, ## indicates *p* < 0.01, and ### indicates *p* < 0.001 when compared to the HFD group. HFD, high-fat diet; HFD+IL, HFD+IJE 0.25% of diet; HFD+IH, HFD+IJE 0.5% of diet; N, normal diet.

We investigated whether IJE can improve HFD-induced decline in exercise capacity. The grip strength and treadmill tests were used to measure exercise abilities. Grip strength was only slightly enhanced in the HFD+IL group (Figure 4A). Furthermore, the treadmill test showed that the distance ran by the mice was enhanced by IJE, especially in the HFD+IL group (Figure 4B). Isoforms of MHC in the skeletal muscle are markers of fiber type delineation. Specifically, MHC I and IIa influence endurance capacity [19,20]. In order to investigate the effect of IJE on muscle fiber type, we compared the mRNA levels of the MHC isoforms of quadriceps and gastrocnemius in mice treated with or without IJE. The mRNA expression of *Myh7* and *Myh2* increased in the group administered low-dose IJE (Figure 4C,D), which was similar to the exercise abilities. Our results also showed that AMPK activity of quadriceps and gastrocnemius was higher in the HFD + IL group than it was in the HFD group (Figure 4E,F). Collectively, the results indicate that IJE can enhance endurance performance by enhancing the mRNA expression of *Myh7* and *Myh2* and the protein level of phosphorylated AMPK.

### 3.5. Effects of IJE Compounds, Luteolin and 1β-Hydroxyalantolactone (1β-HA), on Adipogenic Differentiation and Myogenesis

**Figure 5:**
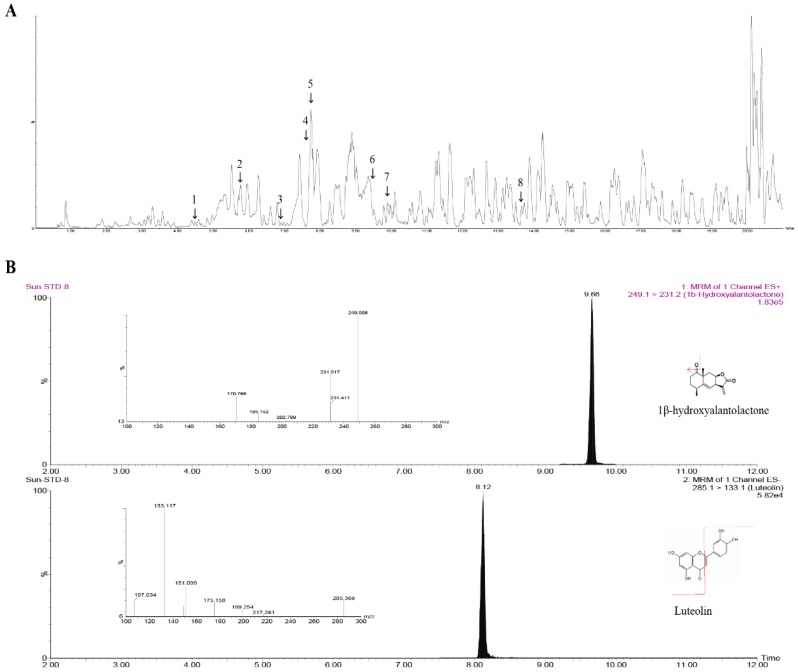
Liquid chromatography–mass spectrometry (LC–MS/MS) analysis of IJE (**A**) chromatogram of IJE, (1) Heliespirone A, (2) 3-Ketoapotrichothecene, (3) O-Formyloreadone, (4) 1-Hydroxyepiacorone, (5) Luteolin, (6) 1β-Hydroxyalantolactone, (7) Heliannuol B, (8) Dihydrocumambrin. (**B**) LC–MS/MS analysis of luteolin and 1β-hydroxyalantolactone (1β-HA) within IJE. Fragmentation pattern spectrum of 1β-HA and luteolin.

We analyzed the putative compounds in IJE by liquid chromatography–mass spectrometry (LC–MS/MS) in positive mode and identified the presence of several compounds (Figure 5A, Table 3). We examined the compounds of IJE based on the results and selected two candidate compounds, luteolin and 1β-HA. These compounds were confirmed based on the accurate masses and retention time compared with reference compounds. Also, we analyzed the amount of the compounds and revealed that IJE contain 2.71 mg/g of luteolin and 1.04 mg/g of 1β-HA (Figure 5B, Table 4).

**Figure 6:**
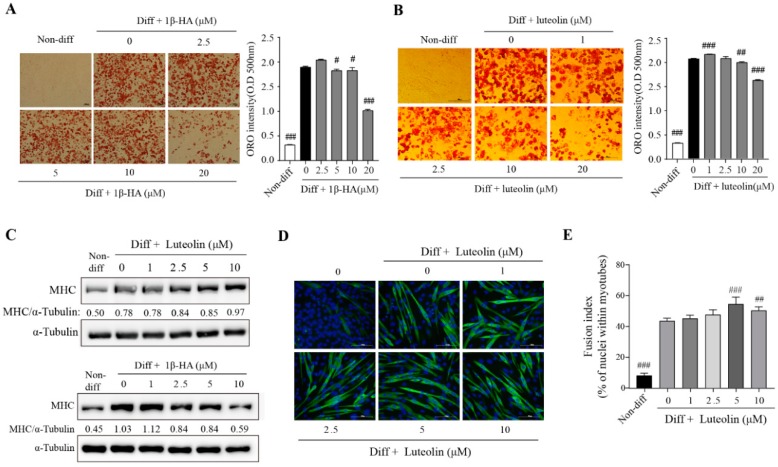
Effects of Luteolin and 1β-hydroxyalantolactone (1β-HA), active compounds of IJE, on adipogenic differentiation and myogenesis in 3T3-L1 and C2C12 cells. Inhibitory effects of (**A**) 1β-HA and (**B**) luteolin on 3T3-L1 adipogenic differentiation were measured by Oil Red O staining (scale bar: 100 μm). (**C**) Myosin heavy chain (MHC) protein level in differentiated C2C12 cells treated with luteolin (0–10 μM) or 1β-HA (0–10 μM). (**D**) Immunostaining of MHC in luteolin-treated C2C12 cells (scale bar: 100 μm). (**E**) Fusion index of luteolin-treated C2C12 cells. Data are presented as mean ± SD of triplicate experiments. #, ##, and ### indicate *p* < 0.05, 0.01, and 0.001, respectively, when compared to the differentiated cells treated without 1β-HA or luteolin. Diff, Differentiation.

We investigated the effects of both compound on the differentiation of 3T3-L1 and C2C12 cells. The results showed that 1β-HA had a considerable inhibitory effect on the adipogenic differentiation of 3T3-L1 cells starting from a concentration of 20 μM (Figure 6A). Luteolin also dose-dependently inhibited 3T3-L1 differentiation into adipocytes starting from a concentration of 10 μM (Figure 6B).

These finding indicate that luteolin and 1β-HA have inhibitory effects on lipid accumulation in adipocytes. The effects of luteolin and 1β-HA on myogenesis in C2C12 cells were also investigated. C2C12 cells were differentiated with or without luteolin or 1β-HA, after which the protein level of MHC was measured. Immunoblot showed that luteolin increased the protein level; however, 1β-HA had no effect on MHC level (Figure 6C). Next, immunostaining of the differentiated cells with an anti-MHC antibody, the number of nuclei in the myotubes was counted. The results showed that luteolin increased fusion index and the number of myotubes in the differentiated C2C12 cells (Figure 6D,E). Taken together, our results indicated that luteolin and 1β-HA, the compounds of IJE, affected myogenic and adipogenic differentiation respectively, and the effects may contribute to alleviate obesity and enhance endurance performance.

## 4. Discussion

In this study, we first examined whether IJE could potentially inhibit obesity through in vitro assay. Although IJE treatment at a concentration of 50 μg/mL significantly inhibited the adipocyte differentiation of 3T3-L1 preadipocyte and the expressions of PPARγ mRNA and protein but the effect was weak, whereas IJE treatment at 100 μg/mL strongly inhibited adipocyte differentiation by 80% via suppresses of PPARγ and C/EBPα, which are known to involve in the adipogenesis. As a result of being in vitro, IJE was expected to prevent obesity through downregulation of adipogenesis and lipogenesis.

Next, we induced obesity in the mice by feeding them HFD for 9 weeks. HFD increased body weight and caused abnormal lipid accumulation in the liver and adipose tissue; however, these were significantly inhibited by IJE. Obesity induces adipogenesis and lipogenesis in the adipose tissue and the liver. In other words, inhibition of adipogenesis and lipogenesis in the liver and adipose tissue shows weight loss [21]. HFD increased adipogenic genes such as of *Fas*, *aP2*, and *Pparγ*; however, these were reduced by IJE. These results are consistent with the results of the 3T3-L1 experiment. Furthermore, the mRNA expression levels of *Scd1*, *Srebp-1c*, and *Cd36*, which mediate fatty acid intake and lipid accumulation, were also decreased in the liver by IJE. However, there was no significant difference in body weight, liver lipids, and fat size between IJE 0.25 and 0.5% in vivo. The expression of lipogenesis and adipogenesis-related genes were inhibited by IJE administration dose-dependently, but this difference was not reflected in the obesity phenotype.

In addition, IJE increased muscle weight in a dose-dependent manner. Obesity not only causes muscle atrophy but also decreases muscle function [22,23]. IJE decreased body weight gain by inhibiting lipid accumulation in the liver and adipose tissue without reducing muscle weight. We also found that IJE improves endurance exercise capacity. Previous studies have shown that obesity reduces exercise capacity [24,25]. Therefore, we tested grip strength and endurance capacity by performing a treadmill test. Our results showed significant reduction in exercise capacity in the HFD group; however, exercise capacity was restored by IJE without any exercise training. These results suggest that IJE improves muscle exercise capacity, especially endurance, and inhibits obesity. Our results showed better effects of IJE at the low dose tested in endurance capacity. Natural product-derived compounds can mediate signal transduction via interaction with various cellular molecules [26]. These compounds are not always expressed in a concentration-dependent manner in vivo because they bind to intracellular molecules in a single or biotransformation or aggregated forms in vivo [27,28] and thus are difficult to explain without clearly interpreting how these compounds act in vivo.

Improvement in endurance is important for sustained supply of energy and resistance to fatigue, and it has been reported that slow muscle of fiber type is involved [29]. Muscle fiber type I has a high mitochondria content and is known to enhance endurance capacity [20]. In the human body, the level of muscle fiber type I is relatively decreased in obese people [30]. In this study, the mRNA levels of *Myh7* and *Myh2* in the skeletal muscles were increased in the HFD+IL group. Thus, IJE had an effect-enhanced endurance capacity by changing muscle fiber type. Stimulation of AMPK activity has been associated with increased exercise capacity [31]. Therefore, AMPK activity can be used to improve endurance. Our results showed that the AMPK level was increased in the IJE-treated mice. Additionally, IJE influenced the conversion of muscle fiber type and activated AMPK, thereby improving endurance. The increase in AMPK activity at a low concentration of IJE was consistent with endurance capacity.

The compounds in *I. japonica* have been analyzed [12,14]; however, the bioactivities of the compounds are not yet fully understood. Previous studies suggested that luteolin and 1β-HA in *I. japonica* extract have bioactivities such as anti-adipogenesis and anti-inflammatory effect respectively [32,33]. However subsequent studies about other activities of these compounds have not been conducted. Our results confirmed that luteolin inhibits 3T3-L1 adipogenic differentiation consistent with those of a previous study [33]. Furthermore, our results showed that the fusion index and the protein level of MHC increased in the differentiated C2C12 cells that were treated with luteolin. This indicates that luteolin improves myogenic differentiation in C2C12 myoblast cells. Luteolin has been reported to reduce cancer-induced skeletal muscle atrophy by increasing muscle weight [34]. Therefore, it seems that luteolin has a potential for improvement of obesity-induced muscle atrophy. Also, our results show a significant inhibitory effect of 1β-HA on 3T3-L1 adipogenic differentiation, but not myogenic differentiation. Collectively, our findings show that luteolin alleviates obesity and enhances endurance capacity. In addition, 1β-HA can be used to inhibit abnormal lipid accumulation.

## 5. Conclusions

In this study, we demonstrated that IJE treatment improves obesity and endurance capacity in HFD-induced obese mice. Our results indicated that IJE can prevent abnormal lipid accumulation in vitro and in vivo by regulating adipogenesis and lipogenesis-related gene expression. Also, it can enhance endurance capacity that is reduced by HFD by increasing AMPK activity and interchanging muscle fiber type. Additionally, our study identified effects of the compounds of IJE, 1β-HA and luteolin on anti-adipogenesis and myogenesis, respectively. Therefore, IJE is expected to be useful to obesity people, and for that, follow-up studies are needed.

## Figures and Tables

**Table 1:** Primer sequences.

Gene	Forward Primer (5′→3′)	Reverse Primer (5′→3′)
*C/ebpα*	CAAGAACAGCAACGAGTACCG	GTCACTGGTCAACTCCAGCAC
*Ppar* *γ*	TCGCTGATGCACTGCCTATG	GAGAGGTCCACAGAGCTGATT
*Fas*	GGAGGTGGTGATAGCCGGTAT	TGGGTAATCCATAGAGCCCAG
*aP2*	CCGCAGACGACAGGA	CTCATGCCCTTTCATAAACT
*Cd36*	ATGGGCTGTGATCGGAACTG	GTCTTCCCAATAAGCATGTCTCC
*Scd1*	TTCTTGCGATACACTCTGGTGC	CGGGATTGAATGTTCTTGTCGT
*Srebp-1c*	TGGATTGCACATTTGAAGACAT	GCCAGAGAAGCAGAAGAG
*Myh7*	CTCAAGCTGCTCAGCAATCTATTT	GGAGCGCAAGTTTGTCATAAGT
*Myh2*	AAGCGAAGAGTAAGGCTGTC	GTGATTGCTTGCAAAGGAAC
*Myh4*	CACCTGGACGATGCTCTCAGA	GCTCTTGCTCGGCCACTCT
*Rn18s*	CTCAACACGGGAAACCTCAC	CGCTCCACCAACTAAGAACG
*Actb*	GCAGGAGTACGATGAGTCCG	ACGCAGCTCAGTAACAGTCC

*C/ebpα*, CCAAT-enhancer-binding protein α; *Pparγ*, peroxisome proliferator-activated receptor γ; *Fas*, fatty acid synthase; *aP2*, adipocyte protein 2; *Cd36*, cluster of differentiation 36; *Scd1*, stearoyl-CoA desaturase-1; *Srebf1*, Sterol regulatory element-binding protein 1; Myh7, myosin heavy chain Ⅰ; *Myh2*, myosin heavy chain IIa; *Myh4*, myosin heavy chain IIb.

**Table 2:** Diet compositions.

Ingredient (g/kg)	N	HFD	HFD+IL	HFD+IH
Casein	200	200	200	200
Corn oil	50	50	50	50
IJE	0	0	2.5	5
Lard	0	200	200	200
Cholesterol	0	5	5	5
Corn starch	350	145	145	145
Sucrose	300	300	300	300
Cellulose	50	50	50	50
Mineral	35	35	35	35
Vitamin	10	10	10	10
Methionine	3	3	3	3
Choline bitartrate	2	2	2	2

N, normal diet; HFD, high-fat diet; HFD+IL, HFD+IJE 0.25% of diet; HFD+IH, HFD+IJE 0.5% of diet.

**Table 3:** Identification of chemical ingredients of IJE using LC–MS/MS.

No.	Molecular Weight	Molecular Formula	Molecular Weight [M+H]^+^	Actual Mass	Mass Error	Retention Time (min)	Fragment (By. HMDB)
1	265.1459	C15H20O4	265.144	265.1427	−1.3	4.5	249, 231, 163
2	250.3	C15H22O3	251.1647	251.164	−0.7	5.8	251, 235, 215, 205, 147
3	264.1362	C15H24O3	265.1427	265.1427	−1.3	6.9	247, 233, 201, 187, 173
4	252.173	C15H24O3	253.1735	253.1735	−6.9	7.7	235, 223, 211, 195, 183
5	286.0477	C15H10O6	287.0572	287.0572	1.6	7.75	287, 255, 153
6	248.3	C15H20O3	249.1487	249.1487	−0.4	9.3	249, 231, 213, 203, 195
7	248.322	C15H20O3	249.1487	249.1487	−0.4	9.9	233, 231, 215, 213, 143
8	308.16	C17H24O5	309.1702	309.1738	3.6	13.6	291, 275, 185, 145

**Table 4:** Quantification of luteolin and 1-hydroxyalantolactone in *IJE.*

Compounds	IJE (mg/g)	*R* ^2^	Linear Rang (μg/mL)	LOQ (μg/mL)	LOD (μg/mL)
Luteolin	2.71 ± 0.35	0.9969	0.5–10	0.28	0.08
1β-hydroxyalantolactone	1.04 ± 0.01	0.999	0.5–10	1.08	0.33

LOD, limit of detection; LOQ, limit of quantification. Data are presented mean ± SD.
